# Vascular Involvement in Emphysematous Pyelonephritis

**DOI:** 10.1155/criu/1636315

**Published:** 2026-03-17

**Authors:** Dana Greenberg, Ronen Rub, Rami Mattar, Yoav Avidor

**Affiliations:** ^1^ Urology Department, Hillel Yaffe Medical Center, Hadera, Israel, hy.health.gov.il

**Keywords:** diabetes mellitus, emphysematous pyelonephritis, urinary tract infection

## Abstract

Emphysematous pyelonephritis is a life‐threatening, rapidly progressive necrotizing infection, characterized by the presence of gas in the affected tissues. Following a rare emphysematous pyelonephritis case which involved gas in the kidney and inferior vena cava vessels, we aimed to review the literature related to diagnosis and treatment. The extension of gas into adjacent vascular structures, though rare, may represent a more fulminant diseases state and warrants particular clinical attention. This case highlights the importance of timely diagnosis and intervention in emphysematous pyelonephritis.

## 1. Introduction

Emphysematous pyelonephritis (EPN) occurs when gas‐forming pathogens cause a necrotizing infection of kidney parenchyma and perirenal tissues. One or more of a few predisposing background conditions is almost always present, most notably diabetes mellitus (DM). The condition often presents as a urologic emergency. Greater awareness of EPN has facilitated earlier and more aggressive treatment, contributing to a decline in mortality rates. Nevertheless, the mortality rate of EPN is significant if not managed appropriately.

The extension of gas into the adjacent large vessels such as the inferior vena cava (IVC) is a rare manifestation of EPN which has ominous implications. In this report, we present one such case, review the broader literature on EPN, and highlight the recent understanding on optimal diagnosis and management. Beyond its rarity, vascular gas extension in EPN remains poorly characterized in terms of pathophysiology and prognostic significance. The present case is aimed at highlighting venous gas involvement as a potential marker of advanced disease severity rather than an incidental radiologic finding.

## 2. Case Report

A 52‐year‐old woman with a medical history noteworthy for uncontrolled DM, asthma, and smoking presented with generalized weakness, persistent and dull right‐sided flank pain, and chills without fever. She reported a loss of appetite and had not taken her routine medication during the prior 3 days.

On admission, she presented with a temperature of 36°C, tachycardia with a pulse rate of 123 beats per minute, and a blood pressure of 88/46 mmHg. On examination, the abdomen was soft and nontender, with no palpable masses or organomegaly. Tenderness in the right flank was found. Laboratory findings were notable for a white blood cell count of 10.4/*μ*L, a glucose level of 825 mg/dL, a creatinine level of 2.7 mg/dL, beta‐hydroxybutyric acid of 0.7 mmol/L, blood pH of 7.322, and CRP level of 556 mg/L. Urinalysis demonstrated 250 white blood cells and 500 red blood cells. Urine culture taken on admission was later positive for *Escherichia coli*.

Contrast‐enhanced computed tomographic (CT) scan demonstrated enlarged right kidney with perinephric stranding, air in the pelvicalyceal system, parenchyma, and throughout Gerota′s fascia. Additionally, a gas bubble was observed in the right renal vein, extending into the IVC (Figure 1a and Figure 2). She was admitted in septic condition to the intensive care unit and started on intravenous fluids, broad‐spectrum antibiotics, norepinephrine, and insulin.

Figure 1(a) Abdominal CT scan showing air in the right kidney parenchyma and in the IVC (yellow arrow), coronal view. (b) Follow‐up CT after stent insertion. Note the disappearance of the gas in the IVC (inferior vena cava). AO (abdominal aorta).

**Figure:**
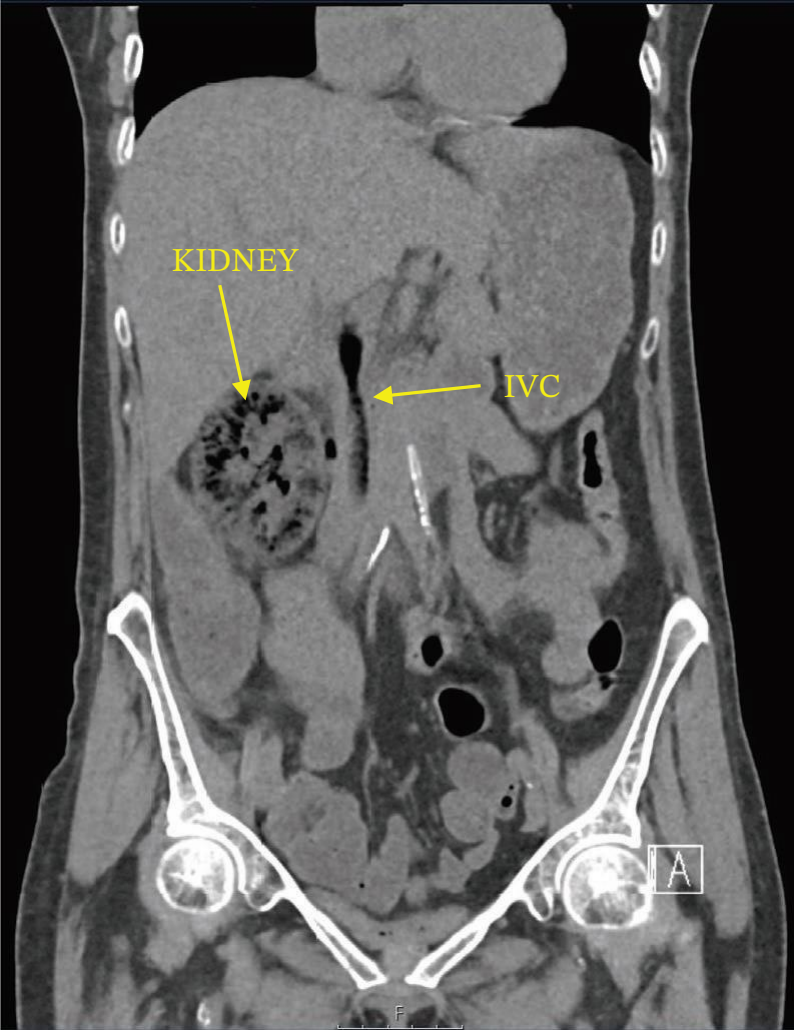
(a)

**Figure:**
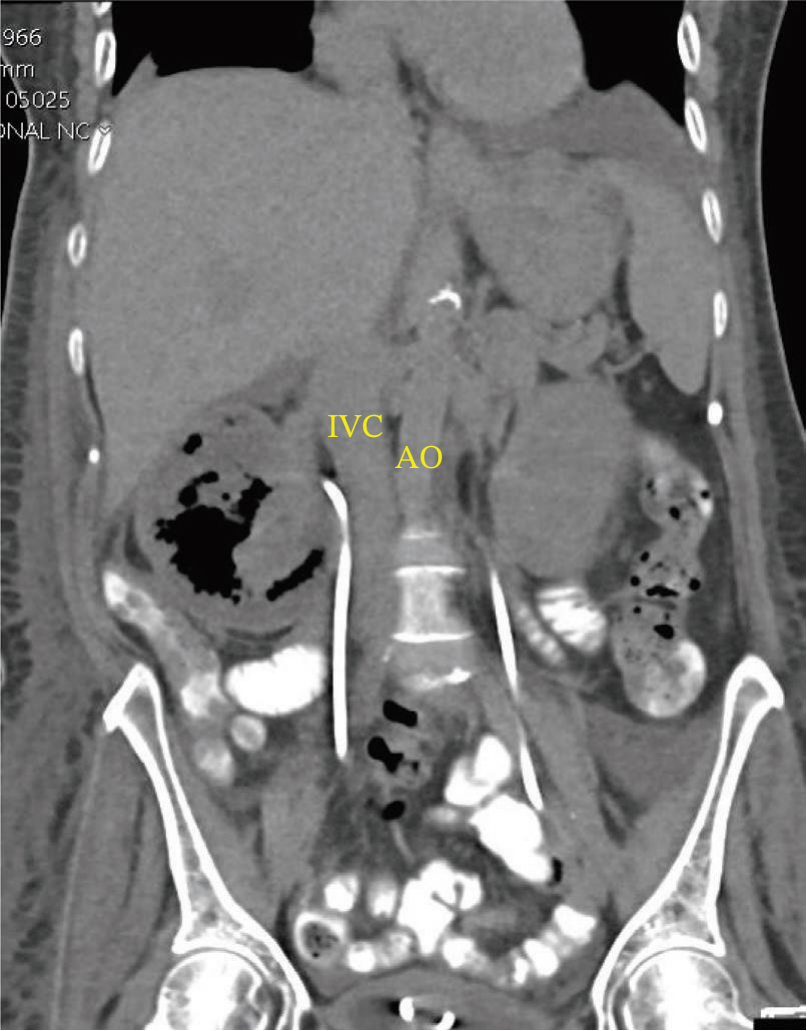
(b)

Figure 2(a) Axial and (b) coronal views of abdominal CT demonstrating a marked difference between the two kidneys. The left kidney appears normal, whereas the right kidney is distended and contains extensive intraparenchymal gas.

**Figure:**
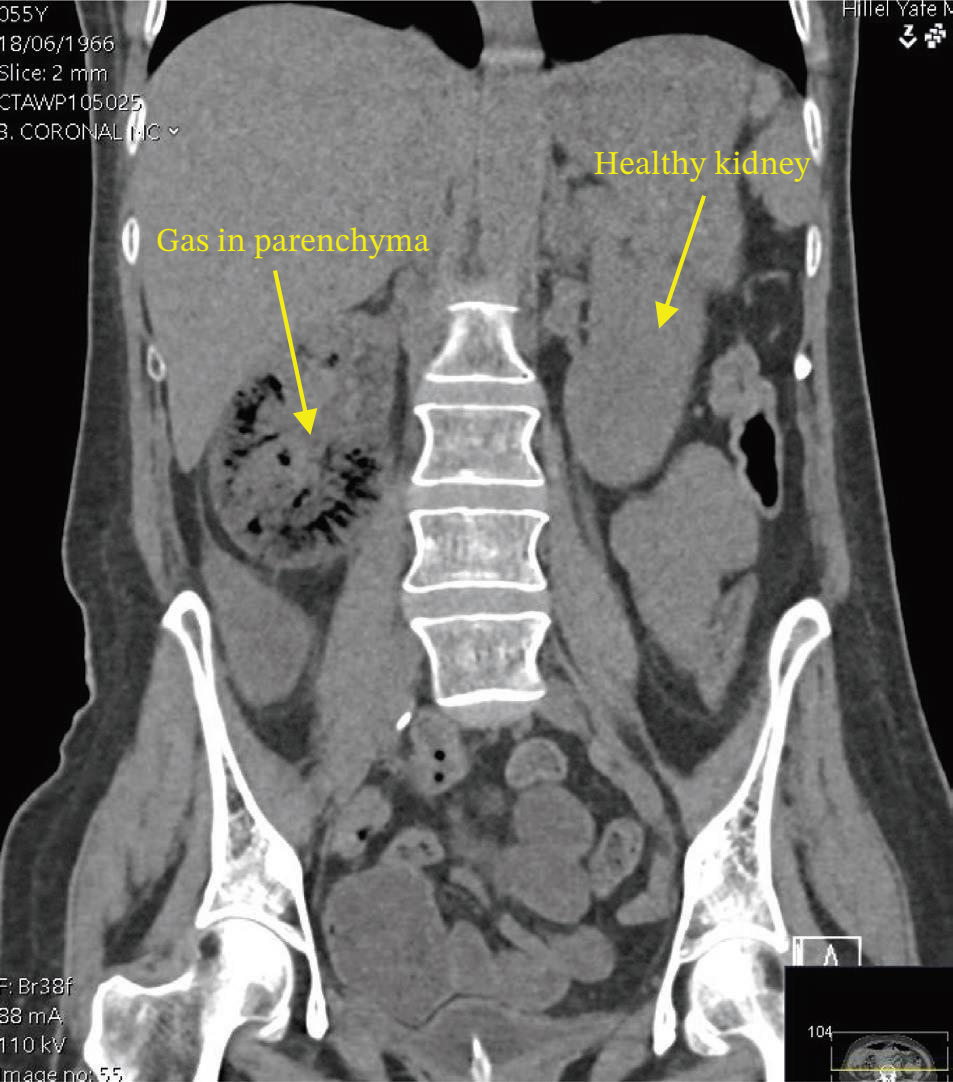
(a)

**Figure:**
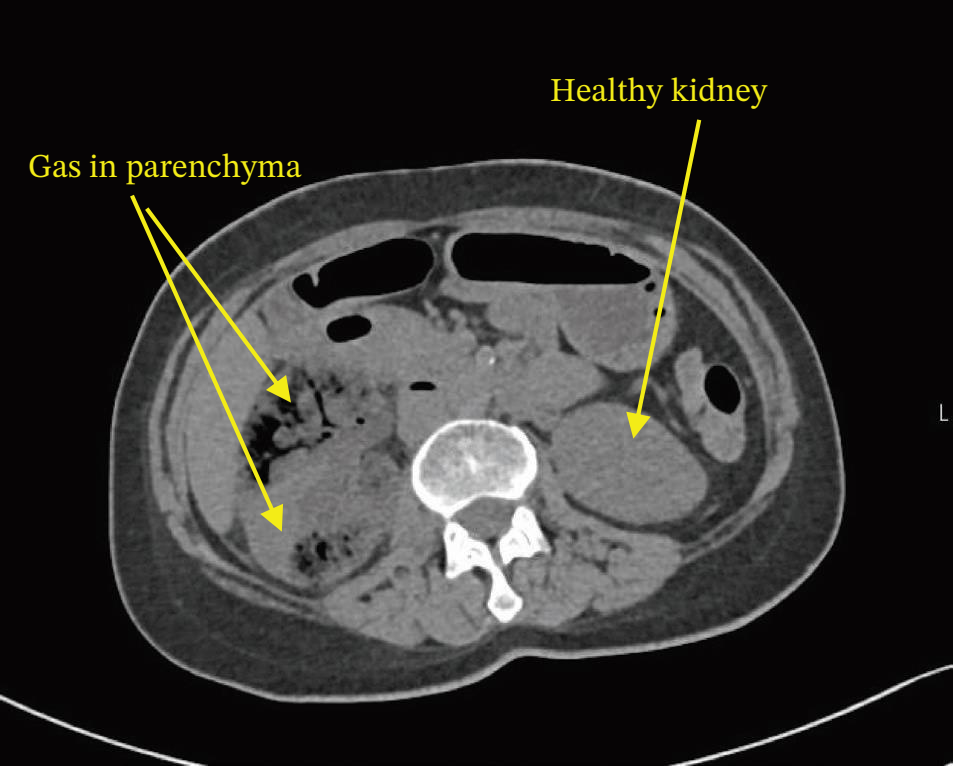
(b)

Following patient stabilization, a double J stent was successfully inserted to the right and left kidneys, yielding a significant amount of purulent urine from the affected kidney. A urine culture obtained from this kidney yielded *E. coli*. Postoperatively, she remained on mechanical ventilation. A CT scan conducted 72 h after the procedure did not capture the gas in the IVC, suggesting improvement of the emphysematous infection (Figure 1b). The patient also resumed independent breathing 5 days after the surgery, her creatinine level normalized to 0.8 mg/dL, and her glucose level declined to 137 mg/dL. She regained consciousness but exhibited signs of aphasia, with no verbal communication evident. Brain magnetic resonance imaging and CT scan which were performed to rule out a cerebrovascular accident revealed a pontine lesion compatible with a new ischemic episode. Four weeks after her initial admission, the patient experienced sudden cardiac arrest and passed.

## 3. Discussion

EPN is a life‐threatening infection of the renal parenchyma which is caused by gas‐forming bacteria [1]. Mortality was originally reported in up to 90% of cases [2], and while still high, has improved to 13%–19% [3–5], thanks to better treatment most notably by percutaneous drainage (PCD) [6].

Approximately 90% of patients with EPN have underlying diabetes [7]. Additional predisposing conditions include immunosuppression, obstructive uropathy, and hypertension [1–2]. Women are affected in 67%–95% of cases [1], a predominance which is likely a result of an increased susceptibility to urinary tract infections [1, 7]. For unclear reasons, EPN is more commonly observed in the left kidney, occurring in approximately 55%–65% of cases. The right kidney is affected in 25%–35% of cases, with approximately 5% of cases exhibiting bilateral involvement [2, 7–8].

Huang and Tseng [1] argued that EPN is more common than originally considered, likely due to the increasing prevalence of diabetes. In our view, rising awareness to EPN and the more common use of advanced imaging studies have undoubtedly also contributed to more frequent diagnosis.

In EPN, gas may be found in the ureters (emphysematous ureteritis), calyces and pelvis (emphysematous pyelitis), and in the bladder (emphysematous cystitis). Gas typically originates from three main sources. First, as in the case at hand, it can be produced by bacteria within the urinary tract. Second, gas may enter the urinary tract as a result of a fistula connecting a hollow viscous organ. Finally, atmospheric gas may be introduced during diagnostic procedures such as cystoscopy or following trauma [7].

In 1941, Gillies and Flocks [9] described three factors which are essential for gas formation to occur in the kidney: urinary tract obstruction, uncontrolled DM, and the presence of gas‐producing organisms. Yet, while true in most cases, more recent literature has shown that EPN may occur even in the absence of urinary obstruction, or more rarely even in the absence of DM [7].

Facultative *E. coli* is the most common gram‐negative pathogen isolated in cases of EPN, accounting for over half the cases, followed by *Klebsiella* (20%–24%), *Proteus* (5%–18%), *Pseudomonas* (5%), and *Enterococcus* (14%) [4]. *Candida* species have also been detected in some cases [10]. Polymicrobial infections are founds in 4%–24% of patients [1, 7–8].

Our understanding of the biochemistry of gas formation is based on analysis of gas obtained from patients who underwent nephrectomy. Yang and Shen, in their elegant study, performed gas chromatographic analysis and were thus able to provide concentrations of individual gas molecules in the infected gas bubbles [11]. These studies validated the hypothesis that the biochemistry of gas formation is connected to bacterial fermentation of glucose via the glycolytic metabolic pathways, which results in the production of carbon dioxide and hydrogen gases. Trace amounts of methane and the nitrogen‐containing ammonia have also been found, most likely as a result of degradation of necrotic tissue and fermentation of amino acids [12–13]. Impaired tissue perfusion and a compromised immune response, which occur in, but are not limited to, DM provide additional fuel to the gas‐producing process, explaining the less common presentations in nondiabetic patients [14].

On admission, patients usually display fever, flank or back pain, dysuria, nausea, vomiting, renal failure, and hyperglycemia. Cases where impaired consciousness, acute renal failure, thrombocytopenia, or sepsis are already present upon initial presentation are associated with increased mortality [1, 7, 10].

CT scan is the most sensitive imaging modality to detect the gas and demonstrate the extent of disease [1]. Michaeli et al. created a useful imaging‐based EPN classification system which was later further enhanced by Huang and Tseng [1, 7]. The system includes four classes of increasing severity, whereby gas confined to the collecting system is the least severe form of EPN, followed by gas in the parenchyma, perinephric, and pararenal spaces. Bilateral EPN, or EPN in a solitary kidney, add to the severity score (Table 1).

**Table 1:** Emphysematous pyelonephritis (EPN) classification by Huang and Tseng.

Class	Description
Class I	Gas in collecting system only
Class II	Parenchymal gas only
Class IIIa	Extension of gas into perinephric space
Class IIIb	Extension of gas into pararenal space
Class IV	EPN in solitary kidney, or bilateral disease

The rationale underlying the Michaeli and the Huang and Tseng classification approach is that imaging‐based evidence of expansion of gas beyond the nephric tissue represents more severe infection and worse prognosis. In fact, since then, a review of approximately 400 EPN patients by Trujillo‐Santamaría et al. corroborated the notion that a higher Huang–Tseng class correlates with worse prognosis and higher mortality [4]. Of note, a significant correlation was also found between a high degree of renal parenchymal gas involvement and worse prognosis and mortality. However, interestingly, Type 4 EPN was associated with similar mortality as Type 3a and lower mortality than Type 3b; this may imply that the systemic impact of Type 3b EPN may portends a more ominous disease than that of EPN in bilateral or a solitary kidney.

Around the publication of the Huang–Tseng criteria, a number of groups investigated clinical prognostic indicators in EPN in order to optimize decision‐making and facilitate aggressive treatment and admission to critical care [15], and several prognostic indicators have been suggested [4]. Kapoor et al. reviewed 39 cases of EPN and found that hypotension, serum creatinine > 5.0 mg/dL, hematuria, thrombocytopenia, and altered consciousness were associated with higher mortality [3]. Khaira et al. reviewed 19 cases of EPN and reached similar conclusions, adding disseminated intravascular coagulation (DIC) to the list of poor prognostic factors [16]. In their report, patients with Class 1 and Class 2 EPN had the best prognosis with 100% survival, following treatment with PCD combined with antibiotics. Interestingly, no association has been found between the degree of glycemic control and outcome of EPN; however, we assume that this might be a by‐product of small sample sizes, and in our view, such a connection cannot be ruled out yet. Urinary obstruction has also been implicated with a worse prognosis and mortality [7].

Taken together, not surprisingly, these studies agree that indications of septic shock and systemic disease portend a worse outcome of EPN. Yet, in our view, the Huang–Tseng criteria remains the most effective EPN classification tool but with two exceptions.

First, including the present case, only a handful of cases of EPN complicated by migration of gas to the vascular system have been reported. Gas was detected in either the renal vein, IVC, or hepatic veins [13, 17–18]. The small number of cases may reflect lack of awareness and also makes it difficult to draw conclusions on prognosis of these cases; however, the degree of renal involvement is known to correlate with EPN severity. For example, involvement of more than 50% of the renal parenchyma based on CT predicts the need for nephrectomy and mortality [3–4]. Following this rationale and based on our experience, we believe that the presence of gas in the major vessels may be associated with secretion of more aggressive bacterial toxins and host radical response which are able to break down the vascular tunica layers.

While the presence of vascular gas has been sporadically described, the underlying mechanism remains poorly understood and is rarely discussed in detail. We hypothesize that venous gas involvement represents not merely a radiologic curiosity, but rather a manifestation of advanced disease severity.

As infection progresses, severe inflammation, microvascular ischemia, and tissue necrosis may compromise the integrity of both renal parenchyma and adjacent venous structures. In parallel, the kidney constitutes a relatively confined anatomical space; therefore, gas accumulation within the parenchyma or collecting system may lead to a rapid increase in intrarenal pressure.

This combination of structural venous wall disruption and pressure gradients may facilitate direct translocation of gas into low‐pressure venous channels, initially involving the renal vein and in more extensive cases, extending into the IVC [17].

More bacteriological research might provide us with further insights on the mechanism. In addition, gas in the IVC can in and of itself cause sudden cardio‐respiratory collapse thus adding another dimension of severity [19]. Thus, it is likely that extension of gas into the great vessels, as seen in the case at hand, is an ominous finding which points to increased severity of tissue damage and more fulminant disease.

Second, the realization that pararenal extension is associated with greater mortality than bilateral EPN or EPN in a solitary kidney, which was not known to Huang and Tseng, when their criteria was developed, justifies an update to the classicization so that the EPN type accurately reflects disease severity and prognosis.

Management of EPN requires aggressive fluid resuscitation, antibiotics, glycemic control, and relief of urinary tract obstruction where necessary [6–7]. Antibiotic coverage should be broad and focused on gram‐negative bacteria. Kidney drainage is essential in order to resolve the infection, expedite drainage of pus, relieve the effects of gas pressure and pus on local circulation, and maximize preservation of renal function. Percutaneous nephrostomy (PCN) is preferred over ureteral catheterization and was shown to preserve renal function in the affected kidney in 70% of cases [16]. The PCN stent should stay in place at least until a follow‐up CT done 4–7 days after initial treatment, which indicates that radiographic evidence of EPN has been resolved [20]. Long‐term renal function in these patients depends on the degree of parenchymal loss and coexisting renal disease. Somani et al. reported on preservation of 64%–72% of nephron capacity in EPN patients treated with PCN [6].

Nephrectomy should be considered in very severe cases, for example, patients in septic shock where medical treatment and drainage fail to stabilize the patient [1]. Since most patients with EPN suffer from DM, it is even more desriable to preserve the affected kidney and maintain nephron function as much as possible.

**Figure 3:**
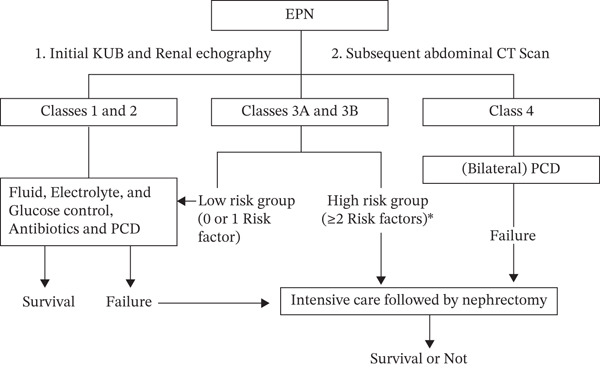
Flowchart for management of EPN according to the clinico‐radiological classification. Asterisk indicated the presence of two or more of the following risk factors: thrombocytopenia, acute renal failure, disturbance of consciousness, and shock. KUB indicated [1].

Huang and Tseng suggested a treatment algorithm based on these principles [1] (Figure 3). Surprisingly, a relatively good outcome with pharmacutical intervention alone was noted [8, 10]. The success rate of combined treatment with PCD and antibiotics was 50% [8]. Additionally, in cases where nephrectomy was deemed necessary, better outcomes were acheived (66%–100%) [1, 8, 10].

**Figure 4:**
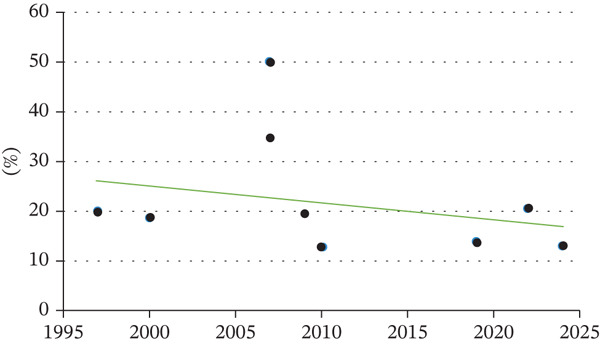
EPN mortality rate trend by years. In green, the statistical trend‐line: 1997 (21), 2000 (4), 2007 (40), 2010 (10, 42), 2019 (29), 2022 (13), and 2024 (12).

The graph (Figure 4) demonstrates a noticeable decline in mortality rates associated with EPN over the past few decades. Each data point represents a different study or case series from various years, with the *x*‐axis representing the years of publication or patient data collection and the *y*‐axis showing the percentage of mortality.

Reported mortality in contemporary reports is approximately 5%–20%. We also see a trend towards more uniform outcomes between the studies, which probably reflects more consistent awareness as the body of evidence on EPN grows and the treatment approach is more mature and accepted.

A downward trend is clearly observable, with mortality rates decreasing from as high as over 50% in the late 1990s to under 10% in more recent years. This trend likely reflects the significant advancements in medical and surgical management of EPN. The introduction of modern surgical techniques, along with improved imaging technologies, has greatly enhanced early detection and the precision of interventions. Consequently, these developments have contributed to better patient outcomes and a marked reduction in mortality.

Multiple authors, including our team, have hypothesized that the decline in mortality can be attributed to these advancements. Early and accurate diagnosis through advanced imaging, combined with timely and effective surgical procedures, has significantly altered the clinical course of EPN, transforming what was once a highly fatal condition into a more manageable one.

Importantly, current EPN classification systems do not account for vascular gas involvement. Our findings suggest that extension of gas into the renal vein or IVC may represent a distinct and clinically meaningful feature of severe EPN that warrants consideration in future prognostic frameworks.

## 4. Conclusion

EPN is a serious complication of pyelonephritis. Most patients suffer from poorly controlled DM, whereby excess glucose provides a more favorable environment for bacterial proliferation. Fluid resuscitation, broad‐spectrum antibiotics, control of blood sugar, and early PCN drainage of the affected kidney are key to achieve optimal outcomes. Improved diagnostics and adherence to these therapeutic principles have resulted in a better outcomes gradual reduction in mortality over the last few decades. Aggressive treatment is also key to preserve kidney function after resolution of the infection. Failure to stabilize the patient may result in shock and often require nephrectomy.

EPN with extension of gas to major veins is an ominous sign which should alert the clinician to a more fulminant disease course. Prospective studies and accumulation of case data will be essential in refining prognostic models and guiding evidence‐based therapeutic decisions in atypical presentations of EPN.

## Funding

No funding was received for this manuscript.

## Consent

The patient described in this case report is deceased. All clinical information presented in the manuscript has been fully anonymized and contains no identifiable personal data. No names, initials, exact dates, or identifying details are included in the text or images. Radiological images were carefully reviewed to ensure that no recognizable metadata or patient identifiers are visible.

## Conflicts of Interest

The authors declare no conflicts of interest.

## Data Availability

The data that support the findings of this study are available from the corresponding author upon reasonable request.
